# Implementation science and the Health Resources and Services Administration’s Ryan White HIV/AIDS Program’s work towards ending the HIV epidemic in the United States

**DOI:** 10.1371/journal.pmed.1003128

**Published:** 2020-11-06

**Authors:** Demetrios Psihopaidas, Stacy M. Cohen, Tanchica West, Latham Avery, Antigone Dempsey, Kim Brown, Corliss Heath, Adan Cajina, Harold Phillips, Steve Young, April Stubbs-Smith, Laura W. Cheever

**Affiliations:** United States Department of Health and Human Services, Health Resources and Services Administration, HIV/AIDS Bureau, Rockville, Maryland, United States of America

## Abstract

Demetrios Psihopaidas and co-authors discuss the implementation science framework of an HIV/AIDS program in the United States.

**Summary points**

- Implementation science has emerged as an essential field for HIV treatment and prevention, promising to maximize the impact of effective intervention strategies to prevent transmission of the virus and to link and retain people with HIV in care.
- The Health Resources and Services Administration’s (HRSA’s) Ryan White HIV/AIDS Program (RWHAP) supports direct medical care and support services for more than half a million people with HIV—more than 50% of all people living with diagnosed HIV in the United States. Through grants to states, counties, cities, and local community-based organizations, the RWHAP supports the coordination and delivery of efficient and effective HIV care, treatment, and support services for low-income people with HIV.
- Since first authorized in 1990, the RWHAP has played a pivotal role in the implementation of effective intervention strategies for people with HIV. RWHAP client outcomes have improved significantly over time, particularly since 2010. However, implementation science frameworks and approaches have created new opportunities to maximize the impact of the RWHAP.
- HRSA’s HIV/AIDS Bureau (HAB), which administers the RWHAP, has developed an approach to support the translation/adaptation of implementation science insights to real-world implementation and evaluation projects; this HAB implementation science approach (HAB IS) is guiding the bureau’s work to maximize the impact of the RWHAP and achieve optimal outcomes for people with HIV along the HIV care continuum.
- In this article, we present HAB IS as a model for other public health agencies and/or faith- and community-based organizations looking to leverage implementation science frameworks and theories to advance their work toward ending the HIV epidemic.
- HAB IS involves 2 core components; the first is rapid implementation—a systematic process for identifying intervention strategies with demonstrated effectiveness at improving outcomes for people with HIV and disseminating them through accessible, multimedia toolkits for rapid replication. The second component is an evaluation framework grounded in implementation science that simultaneously assesses the impact of an intervention strategy on client outcomes (client outcomes), the penetration of an intervention strategy in a specific setting (implementation outcomes), the utility of specific implementation strategies to achieve uptake and integration of the intervention strategy (implementation strategies), and the effect of broader contextual factors that affect implementation (barriers/facilitators).
- By supporting the scale-up of effective intervention strategies to decrease morbidity and mortality and improve health outcomes along the HIV care continuum for people with HIV, implementation science is advancing the work of HRSA HAB and the RWHAP, ultimately bringing us closer to ending the HIV epidemic in the US.

## Introduction

Implementation science has emerged as an essential field for HIV treatment and prevention, providing crucial insights for clinical effectiveness and efficacy trials, bench-to-bedside translation of clinical trial evidence into real-world intervention strategies, and routine program monitoring and evaluation [1,2,3,4,5]. Implementation science research draws theoretical approaches and frameworks from multiple qualitative and quantitative research traditions [5] in the pursuit of identifying “methods to promote the systematic uptake of research findings and other evidence-based practices into routine practice, and, hence, to improve the quality and effectiveness of health services” [6]. While outcomes from this research are increasingly becoming available, translating the findings and adapting the methods to inform real-world implementation and evaluation studies has proven to be challenging [3,5,7,8]. The Health Resources and Services Administration (HRSA) HIV/AIDS Bureau (HAB), which administers HRSA’s Ryan White HIV/AIDS Program (RWHAP), has developed a framework to support this translation/adaptation process, guiding implementation and evaluation projects across the RWHAP. In this paper, we present this framework—the HAB implementation science approach (HAB IS).

Funded at $2.3 billion in 2019, with more than 2,000 providers across the US, the RWHAP delivers a comprehensive system of high-quality HIV care and treatment, including direct medical care and support services for more than half a million people with HIV—more than 50% of all people living with diagnosed HIV in the US [9]. Since 1990, the RHWAP has played a pivotal role in supporting state and county health departments as well as faith- and community-based organizations to implement effective intervention strategies to improve the health and well-being of people with HIV. HRSA HAB has worked with RWHAP-funded grant recipients and providers to develop novel intervention strategies to link and retain clients in high-quality care, particularly for key populations carrying the greatest burden of HIV. Meanwhile, HIV primary care and supportive service providers, many funded by the RWHAP, have also developed and adapted their own innovative intervention strategies to address urgent needs [2].

These program innovations, coupled with developments in biomedical science over the last decade, have resulted in significant improvements in RWHAP client outcomes along the HIV care continuum [10]. Notably, the percentage of clients reaching viral suppression increased from 69.5% in 2010 to 87.1% in 2018 [9]. Outcomes for key populations with historically disparate viral suppression have also significantly improved since 2010 [11]. For example, the disparity between Black or African American clients and White clients reaching viral suppression decreased by nearly half during this period, from a 13.0 percentage point difference in 2010 to a 6.9 percentage point difference in 2018. Additionally, in 2018, 81.1% of transgender women reached viral suppression compared to 62.2% in 2010, an 18.9 percentage point increase [9].

Given these historical successes, support for ongoing RWHAP-funded services and HRSA HAB activities will likely continue to play an important role in improving outcomes and reducing disparities for people with HIV. However, to bring us closer to ending the HIV epidemic in the US, HRSA HAB seeks to maximize the impact of the RHWAP by leveraging insight from implementation science. By describing HAB IS in this paper, we aim to support other public health agencies and community-based organizations that may be similarly trying to strengthen program implementation and evaluation projects. Additionally, we aim to advance implementation science by (1) contributing to the dialogue between scientists and implementers, demonstrating how implementation science can be utilized in real-world settings, and (2) providing a framework through which real-world knowledge can be consistently produced to inform future directions for implementation research.

### Ending the HIV epidemic with implementation science

Biomedical innovations over the past decade have produced very powerful tools that are essential to end the HIV epidemic. People with HIV who take HIV medication daily as prescribed and who reach and maintain an undetectable viral load have effectively no risk of sexually transmitting the virus to an HIV-negative partner [12]. In addition, regimens of antiretroviral drugs known as pre-exposure prophylaxis (PrEP) and post-exposure prophylaxis (PEP) have proven in clinical trials to be effective agents against HIV transmission for HIV-negative people [13,14,15,16]. According to the Centers for Disease Control and Prevention (CDC), when taken daily, PrEP is highly effective for preventing HIV infection [17]. Collectively, effective use of these tools can greatly reduce new HIV infections and bring the US closer to ending the HIV epidemic [18].

With these tools in hand, greater emphasis is now being directed toward implementation by federal agencies, researchers, and direct medical care and support service providers, with the goal of expanding access to treatment for all people with HIV and access to PrEP for all those who need it [18]. But implementation is a complex social process that includes multiple phases [19] and is always embedded in and shaped by the context in which it takes place [20]. These complexities present a host of challenges distinct from those faced by the efficacy and effectiveness randomized controlled trials (RCTs) that gave us contemporary HIV medications. Implementation science emerged in an effort to create frameworks to systematically address these complexities [6].

The questions that implementation science seeks to address have long preoccupied a range of qualitative and quantitative traditions, including health services research, health behavior research, medical sociology, management science, industrial engineering, and organizational science [5,20,21]. Drawing together insights from these disparate fields, implementation research has made significant strides toward improving our ability to address the complexities and challenges of implementation. Influential models generated include RE-AIM [22], the Proctor Model [23], the Consolidated Framework for Implementation Research [20], Precede-Proceed [24], and PRISM [25]; these models support the systematic assessment of implementation processes and outcomes. Scholars have also developed typologies of implementation research study designs that signal how, when, and which contextual factors and other mediating and moderating variables are measured, since traditional RCT designs are not necessarily optimal or appropriate for implementation research [26,27].

Despite these advances, many public health agencies and implementers have experienced challenges in operationalizing available models and concepts from implementation science in the real world [22,27]. Researchers have noted that challenges facing the field continue to be the need for standardized terminology, consistent application of concepts, and abstracted findings across multiple studies to generate new frameworks for research [28,29,30]. Some researchers have observed that there are now so many theoretical approaches for studying implementation that it has become difficult to identify which is best suited for any particular study [31,32,33].

For real-world implementers, another significant challenge is what Geng and colleagues (2017) describe as a tension between rigor and relevance [7]. Rigor has historically been associated with the scientific principles codified in RCTs, where strict study conditions and participation criteria must be maintained, even if such controls reduce direct applicability—or similarity—to the real world. While RCT designs have been essential for drug efficacy trials, when implementation is the goal (e.g., to understand how to scale-up prescribing of medications known to be efficacious), implementers and evaluators have pushed for study designs with greater relevance to real-world contexts [22]. Trying to rigorously account for the messiness of the real world, however, can require elaborate, resource-intensive evaluation frameworks that are hard to replicate. Moreover, studies that are highly tailored to, and therefore relevant for, a specific context may have reduced external validity, hindering the production of generalizable knowledge that could support broader implementation [7].

For HIV prevention and treatment, this tension emerges specifically in relation to identifying and scaling up intervention strategies. To carry out implementation research in HIV, it is necessary to identify intervention strategies with previously demonstrated evidence of effectiveness so that the study can focus on how to achieve successful uptake and integration of the intervention strategy. But deciding which intervention strategies should count as having demonstrated effectiveness becomes a point of contestation. Rigor is typically attributed to intervention strategies with published research evidence of effectiveness. On the other hand, intervention strategies are relevant when they are feasible for implementation and address current priorities. Intervention strategies that are especially innovative and relevant may have shown success locally but may not yet have had time to establish a basis of published research evidence, thereby seeming to have insufficient rigor to be worthy of replicating these intervention strategies at scale. Yet intervention strategies rigorously assessed through RCTs may not provide evidence that is readily relevant to or sufficient for application in the real world [22,27]. And, by the time an intervention strategy has had time for its effectiveness to be demonstrated through published evidence, it may no longer address the most urgent priorities.

This tension notwithstanding, implementation science remains a promising resource for maximizing the successful integration and scale-up of effective intervention strategies to improve outcomes and eliminate disparities for people with HIV. Recent work has demonstrated that it is possible to achieve relevance without sacrificing rigor [35]. Studies have concluded that in order to balance this tension and maximize the impact of implementation science in HIV, stakeholder involvement must be included at all levels of implementation, findings and lessons learned must be disseminated rapidly to ensure real-world relevance, and program evaluation should be ongoing with iterative feedback loops to make mid-implementation adjustments [36,37,38]. These findings align with recent community-driven program and policy recommendations that have emphasized the need for greater flexibility to implement innovative intervention strategies with real-world relevance and increased responsiveness to community feedback in program and policy decision-making [39].

Additionally, implementers can encourage the field to develop in ways that will foster greater relevance if the permeation of implementation science into real-world implementation research is improved [34]. One strategy to achieve both the permeation of implementation science concepts and the production of real-world feedback for the field is to create guidelines that can support the application of implementation science theories, models, and frameworks into implementation research and practice [34]. HAB IS, the approach presented in the sections below, reflects this strategy.

HAB IS offers a framework of guiding principles that can support efforts to use the growing body of available implementation science frameworks and concepts in the RHWAP and other HIV care and treatment settings. HAB IS is not intended to replace any specific implementation science framework but rather to support the use of any framework or theoretical approach. HAB IS is intended to be tailored iteratively to meet the needs of each implementation and evaluation project. In other words, HAB IS reflects the principle of equifinality; that is, each individual project developed within the HAB IS framework is expected to take a unique path to accommodate specific contextual factors, mediating variables, intervention strategies utilized, and other needs while arriving at a similar objective: producing real-world knowledge of how to successfully implement intervention strategies to reduce disparities and improve health outcomes for people with HIV.

## Methods

Building upon a broad effort to advance HIV programming with implementation science, in 2018 HRSA HAB began a close collaboration with the CDC Division of HIV/AIDS Prevention and the National Institutes of Health, National Institute of Mental Health (NIMH) to create a platform for dialogue across federal agencies working to apply implementation science in HIV, establishing the Federal Implementation Science Workgroup (Federal Workgroup). The Federal Workgroup sought to coordinate efforts to define and standardize applications of implementation science across these agencies, as well as to identify the unique needs and role of each agency.

Monthly conference calls brought Federal Workgroup members together to discuss existing and planned projects, present findings and lessons learned, identify areas of need, and to plan panels and workshops at professional meetings and conferences. Throughout 2018 and 2019, members of the Federal Workgroup presented on several implementation science panels, soliciting feedback on our implementation science activities from researchers and community members.

Simultaneously, HRSA HAB established an internal HAB Implementation Science Workgroup (HAB Workgroup) with a diverse group of resident implementation science and RWHAP subject matter experts. The HAB Workgroup provided a forum to define implementation science in the context of the RWHAP and to identify past, current, and future projects utilizing some form of implementation science to support the development of a general framework that could guide a more standardized application of implementation science across the RWHAP.

To achieve this goal, HAB Workgroup members conducted in an extensive literature review to identify key concepts in the field and conducted an environmental scan of the use of implementation science at other federal agencies. The workgroup held monthly consensus meetings to modify existing definitions, concepts, and frameworks, as well as to develop guidance documents to describe implementation science specifically adapted for the RWHAP. Through multiple HRSA HAB-funded projects, the workgroup’s guidance was also codified into quantitative and qualitative evidence rubrics and other crucial tools to support a more standardized application of implementation science across the RWHAP. HAB Workgroup findings and interim products were also regularly presented for feedback at several professional meetings and conferences; the Federal Workgroup also provided a venue to discuss HAB Workgroup activities and objectives to promote complementarity with the activities of other federal agencies.

Through these activities, HAB IS was clarified and codified. Although it is beyond the scope of this paper, the role of HAB IS and its relationship to other federal efforts was also clarified. In the following sections, we describe HAB IS. We then conclude with a discussion of the opportunities and ways to mitigate potential risks of adopting an implementation science framework like the one described here.

## Tailoring implementation science for the RWHAP: Definitions

The following definitions are intended to support implementation efforts specifically within the RWHAP. The definitions may also be useful in other contexts, but we recommend tailoring them to meet local programmatic needs.

### Implementation science

**Fig 1:**
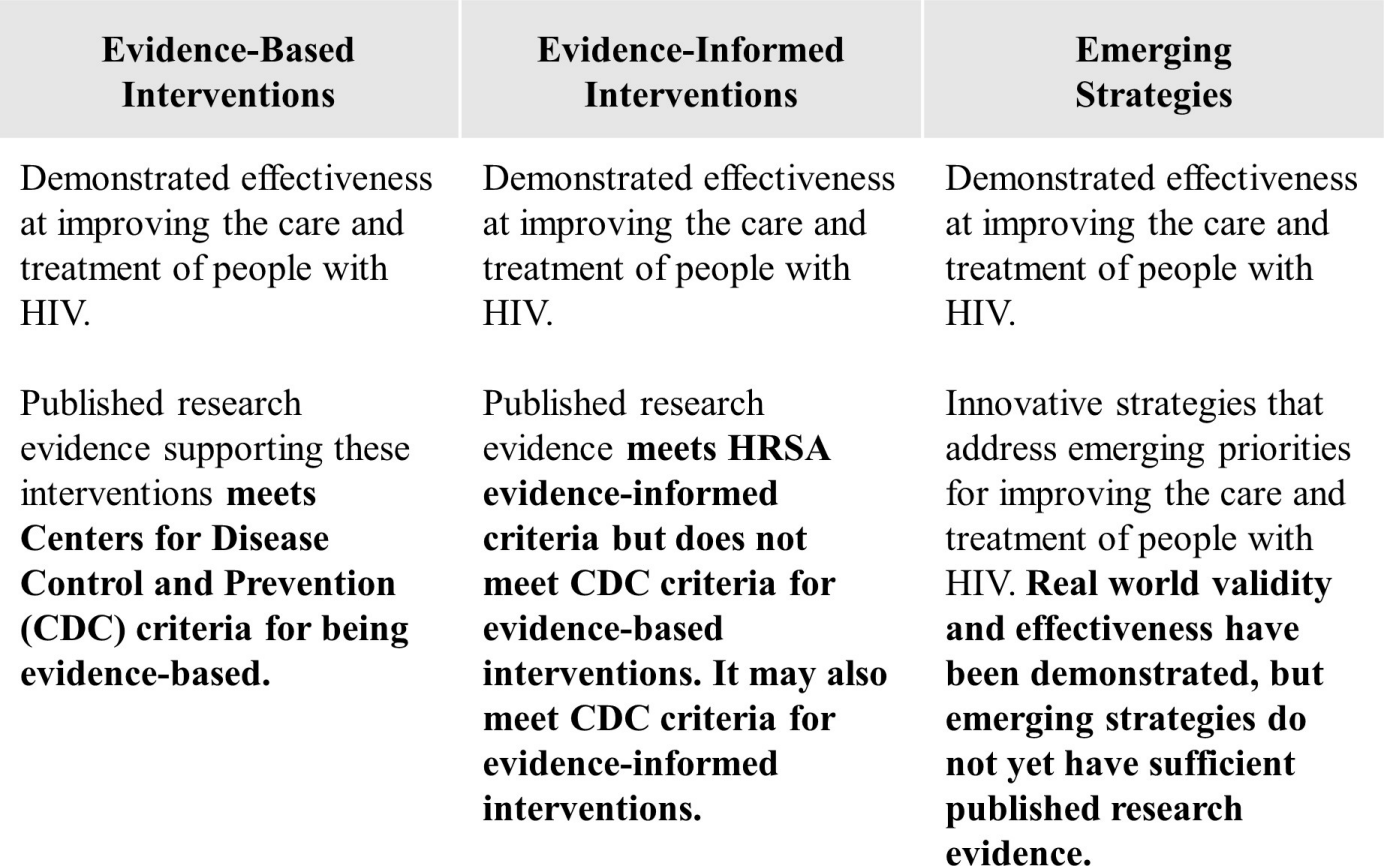
Categories of intervention strategies captured for dissemination by HRSA HAB. HAB, HIV/AIDS Bureau; HRSA, Health Resources and Services Administration.

For the RWHAP, implementation science is the scientific study of methods to promote or improve the systematic uptake of intervention strategies with demonstrated effectiveness into practice, program, and policy. To define “demonstrated effectiveness,” HRSA HAB, in collaboration with the CDC and NIMH, developed 3 categories of intervention strategies for the RWHAP: evidence-based interventions, evidence-informed interventions, and emerging strategies. In Fig 1, we describe the differing types and strength of evidence for these categories of intervention strategies. Intervention strategies can meet HRSA HAB’s criteria for “demonstrated effectiveness” by meeting the criteria of any of these 3 categories.

### Intervention strategies

For the RWHAP, intervention strategies are activities or practices that improve outcomes along the HIV care continuum. Intervention strategies may be simple tools (e.g., alcohol screening and brief intervention) or they may be complex, involving multiple components. Finally, intervention strategies may occur at any level of healthcare, including the system/environment, organizational, group/learning, supervisory, and individual (provider/client) levels.

### Implementation strategies

Implementation strategies are methods or techniques used to enhance the adoption or uptake of intervention strategies in specific settings. While process evaluations have come to be an integral part of evaluation frameworks developed through HRSA’s Special Projects of National Significance (SPNS) program, the implementation strategies concept permits a more systematic evaluation of strategies that promote successful implementation.

### Hybrid studies

While in the purest sense, implementation science is concerned with the successful uptake and integration of a new practice into routine care, all HAB IS projects are “hybrid studies.” Hybrid studies in implementation science combine elements of clinical effectiveness and implementation research to enhance public health impact [40]. HRSA HAB is not only concerned with the uptake and integration of an intervention strategy but also with demonstrating an associated impact on health outcomes, primarily those along the HIV care continuum. These impacts are considered to be associated with the intervention strategy because we cannot control for other factors that may also be influencing these outcomes and/or attenuating the impact of an intervention strategy. This approach is integral to HAB IS as described below.

## Implementation science for the RWHAP: General framework

HAB IS entails 2 core components: rapid implementation and implementation science evaluation. In this section, we provide a general description of each of the steps involved in these core components. In S1 Appendix, we provide a concrete application of each step of each component of the framework to a HAB IS project currently being implemented, the Using Evidence-Informed Interventions to Improve Health Outcomes among People Living with HIV initiative (referred to simply as “E2i”) [41].

### First core component: Rapid implementation

**Fig 2:**
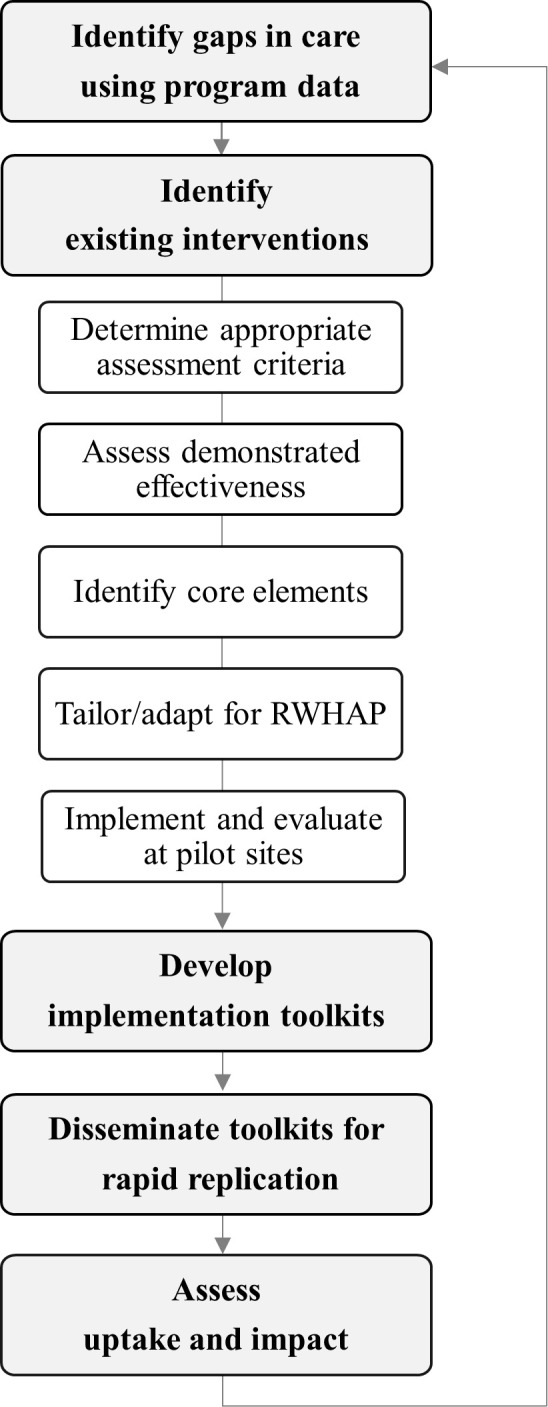
Core component 1: Rapid implementation. RWHAP, Ryan White HIV/AIDS Program.

Fig 2 depicts rapid implementation, HRSA HAB’s organizational process for systematically identifying existing intervention strategies within the 3 categories described earlier. Interventions strategies are identified specifically with the goal of disseminating those found to be impactful for rapid implementation across the RWHAP.

#### Identification of gaps in care using program data

To achieve optimal HIV outcomes for RWHAP clients, and to reach national and program-specific goals, a central part of HRSA HAB’s strategic plan is to advance data dissemination and utilization for program monitoring and quality improvement. HRSA HAB relies extensively on engagement with stakeholders—including HRSA HAB Project Officers, RWHAP-funded grant recipients and providers, and national partners—to inform ongoing efforts to improve data utilization, data visualization and dissemination, and data quality, as well as to make RWHAP data more actionable to strengthen the program and achieve optimal outcomes for our clients. Using the latest and best available program data, strategic focus areas and key subpopulations can be identified.

#### Identification of existing intervention strategies

Utilizing several previous and current HRSA HAB cooperative agreements, extensive literature reviews, technical expert panels, and community advisory boards, HRSA HAB has identified a number of existing intervention strategies with demonstrated effectiveness. To complement this process, a centralized web portal is currently under development, through which users will be able to submit a short narrative describing an innovative intervention strategy, which will then be assessed for inclusion in a comprehensive compilation of catalogued intervention strategies. HRSA HAB will then follow up with the submitter to fully assess the intervention strategy using established evidence assessment criteria and standardized rubrics. Through the portal, RWHAP recipients and providers—as well as non-RWHAP-funded providers, HIV researchers, and other stakeholders—will be able to access a comprehensive compilation of known intervention strategies with demonstrated effectiveness. Information about how specifically to implement these intervention strategies will support RWHAP providers and other HIV care and treatment providers in efforts to address client outcome disparities.

#### Determination of appropriate assessment criteria

**Fig 3:**
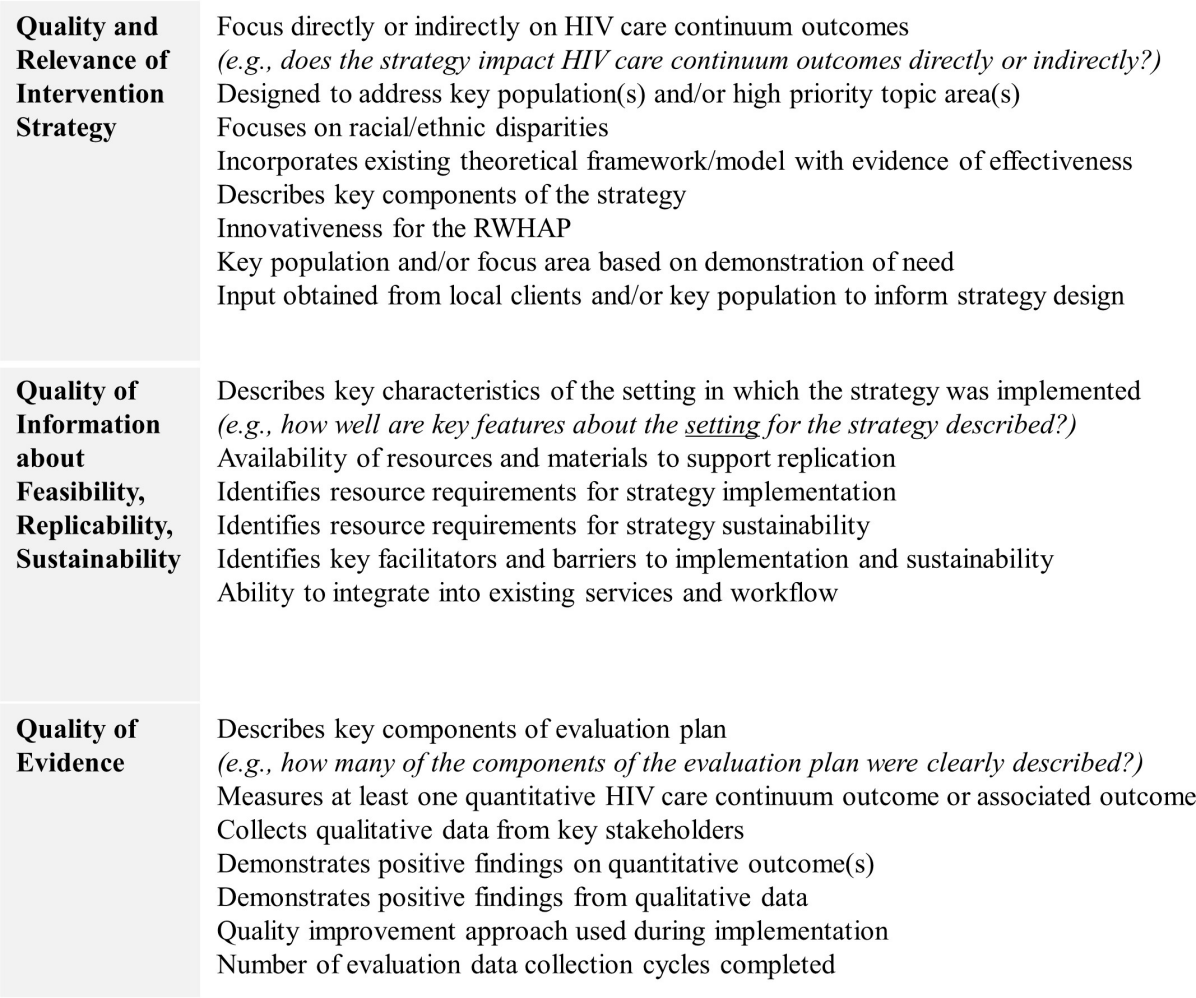
Assessment domains for the identification of emerging strategies. RWHAP, Ryan White HIV/AIDS Program.

In order to screen intervention strategies systematically, HRSA HAB has developed 3 domains that balance strength of available evidence of effectiveness with relevance to the specific context of care and population base served by the RWHAP, as well as the likelihood that the intervention strategy could be rapidly implemented in new settings. Fig 3 depicts these 3 domains.

#### Assessment of demonstrated effectiveness

Following a step-wise logic, intervention strategies with published research evidence will be routed to an evidence assessment rubric. Intervention strategies that have published research evidence will be assessed to determine whether they meet HRSA HAB’s established criteria for evidence-informed or evidence-based interventions. Intervention strategies that do not yet have published research evidence—or if their evidence does not meet the criteria for being evidence informed or evidence based—will be assessed using a distinct rubric for emerging strategies.

#### Identification of core elements

The core elements of an intervention strategy can be defined as the “active ingredients” that are essential to achieving the desired outcomes. These core elements include specific activities, processes, or behaviors that are integral to the intervention strategy (e.g., brief trauma screening) and may also include specific methods or techniques used to implement the intervention strategy. That is, these techniques may include implementation strategies that are also considered to be integral to the intervention strategy (e.g., utilize peer navigators rather than clinicians to deliver the brief trauma screening). Additional implementation strategies and/or activities, processes, or behaviors may later be combined with these core elements in the process of tailoring the intervention strategy for implementation in a specific setting. Differentiating these core elements from customizable components is essential to ensure that local customization is not harming the effectiveness of the intervention strategy. The developers of the intervention strategy may be a helpful resource to achieve this important objective.

#### Tailoring and adapting intervention strategies for the RWHAP

In order to promote rapid implementation in new settings, interventions that have been identified, have demonstrated effectiveness, and are considered to be likely to be rapidly implementable in RWHAP settings still may require specific tailoring or adaptations to make them ready for rapid implementation and to promote sustainability. In particular, specific implementation strategies that are not already part of the core elements of the intervention strategy as described may be added at this stage to provide guidance to RWHAP recipients and providers. Further tailoring may also take place at the local level when the intervention strategy is implemented.

#### Implement and evaluate at pilot sites

Through initiatives such as E2i (described in S1 Appendix), HRSA HAB supports the piloting of identified intervention strategies in RWHAP settings. The goal of these initiatives is to see whether an intervention strategy—determined through our assessment criteria to have demonstrated effectiveness—can be successfully implemented and demonstrate an associated impact on improving outcomes along the HIV care continuum for clients in RWHAP settings. Through the pilot sites, RHWAP providers are able to draw from real-world experience to inform the adaptation/tailoring of the intervention strategy and the development of implementation toolkits or guidance manuals (described below) to support successful replication of the intervention strategy at other RHWAP sites. Pilot sites are provided technical assistance and evaluation data support to determine what is working and what, specifically, is making it work. Lessons learned are also captured for dissemination in the implementation toolkits.

#### Develop implementation toolkits

Implementation toolkits or guidance manuals are how-to manuals that are the central product of projects developed in the HAB IS framework. In order to facilitate rapid implementation, these toolkits include abbreviated information about the intervention strategy, including the core elements (intervention strategies and/or implementation strategies), any recommended adaptations to facilitate implementation in RWHAP settings, tools to perform a simple local evaluation to gauge uptake and impact, case studies of successful implementation efforts, and other guidance. Whereas these toolkits reference and link to supporting documentation—which may include comprehensive manuals describing the intervention strategy, supporting literature, evaluation data, and more—the toolkits themselves are designed to be user-friendly and highly accessible for busy RWHAP recipients and providers who may have identified a gap in care and a relevant intervention strategy to try to address it.

#### Disseminate toolkits for rapid implementation

Toolkits and other resources are centrally disseminated through HRSA HAB’s public-facing site TargetHIV.gov. Although the products of HAB IS projects are intentionally tailored specifically to the RWHAP, they also are expected to be useful for non-RWHAP providers with some additional tailoring.

#### Assess uptake and impact

Through contracts such as the RWHAP Best Practices Compilation [42] and ongoing program monitoring activities, HRSA HAB assesses the uptake and impact of disseminated implementation toolkits. Specifically, HRSA HAB will assess (1) whether toolkits are reaching those who need them the most, (2) when toolkits reach their intended targets, whether they are able to replicate the intervention strategy successfully, (3) whether the toolkits are being accurately interpreted, including a clear understanding of the resources and organizational capacity needed to replicate the intervention strategy successfully, and (4) whether there are specific kinds of technical assistance that are consistently needed to support the replication of an intervention strategy. This information will support future initiatives and toolkit development.

## Second core component: Implementation science evaluation

Implementation science evaluation is the second core component of HAB IS. Although the objectives of implementation science evaluations are not wholly different than monitoring and evaluation in public health programs—that is, they both seek to understand what is working and why—monitoring and evaluation has historically focused more systematic analysis on the services provided rather than barriers/facilitators to implementation. Implementation science evaluations seek to dedicate at least as much effort at systematically assessing these implementation factors. The HAB IS evaluation framework is designed to focus simultaneously on assessing (1) the uptake and integration of intervention strategies; (2) understanding implementation processes, including assessing specific implementation strategies; (3) understanding broader contextual factors affecting implementation; and (4) understanding the impact on client outcomes. For the RWHAP, evaluation study designs may be used to assess individual sites, multiple discrete sites, or linkages across health systems. These evaluations may entail within-group or between-group comparisons.

**Fig 4:**
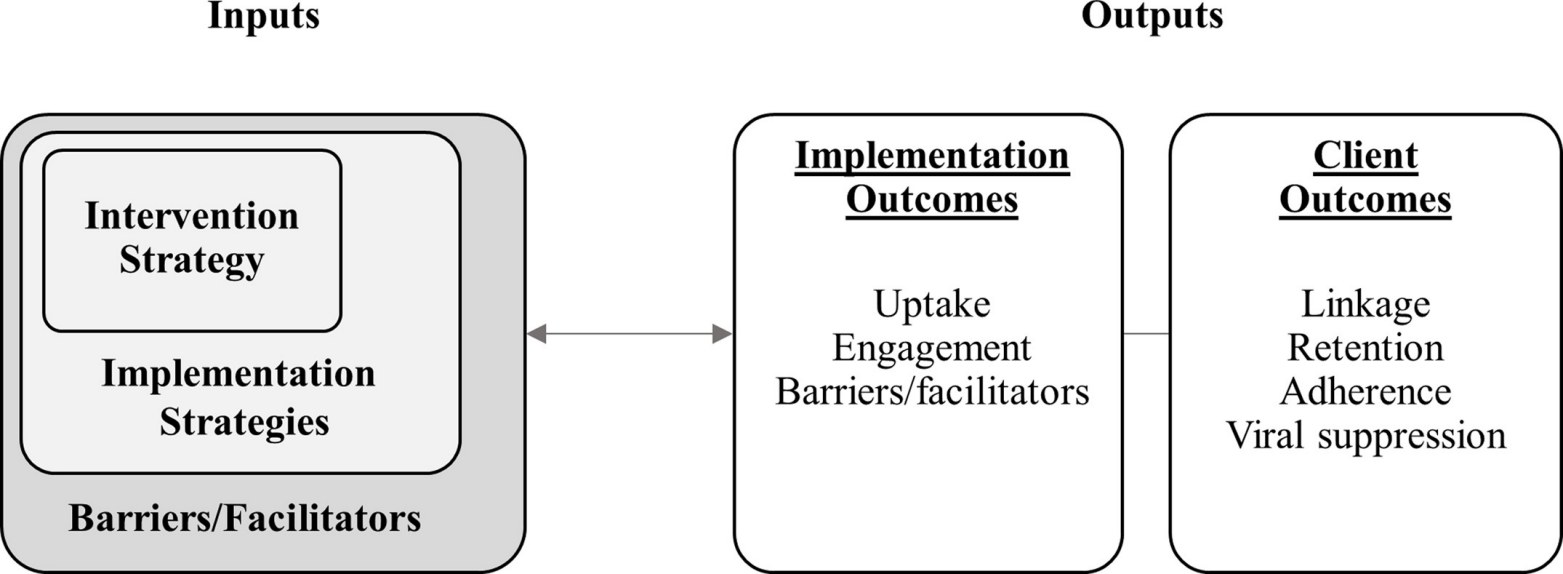
HRSA RWHAP’s implementation science evaluation. HRSA, Health Resources and Services Administration; RWHAP, Ryan White HIV/AIDS Program.

Implementation science evaluation is depicted in Fig 4. This framework includes both process and outcome measures that address the following core questions:

- Was the intervention strategy successfully taken up in the setting?
- What about the context/environment shaped its implementation?
- Were clients successfully engaged in the intervention (implementation outcomes)?
- Were there associated improvements in client outcomes along the HIV care continuum (client outcomes)?

Implementation science evaluation outcomes are defined as follows:

**Implementation outcomes** reflect the effectiveness of the implementation strategies used to promote adoption and use/uptake of the intervention.

- Adoption of the intervention by the target population (e.g., organization leadership, direct care providers)
- Context or environment influencing implementation, either internal (e.g., organizational culture, staffing, resources, procedures) or external (e.g., state or federal law, integration within a health system) to the implementation setting.
- Engagement of the intended client population.

**Client outcomes** reflect the impact of the strategy or intervention on health outcomes for people with HIV along the HIV care continuum (e.g., linkage to care, retention or re-engagement in care, medication adherence, viral suppression).

This general framework is intended to be tailored to meet the specific needs of each evaluation. The evaluation plan for a single clinical implementation of a provider-level intervention strategy, for example, will necessarily look quite different than a statewide implementation of system-level integration of care intervention strategy. Perhaps most importantly, the framework is designed to capture the minimal components of an implementation science evaluation. In resource-limited settings, a minimal framework may be necessary, but in other settings the framework can be expanded to include helpful variables from implementation science, such as “determinants” [20] and “service outcomes” [23].

## Discussion

Advances in HIV science over the past decade coupled with the development of innovative intervention strategies have produced very powerful tools essential for ending the HIV epidemic. To maximize the potential of these tools, HRSA HAB developed HAB IS described here. HAB IS aims to support the systematic identification of innovative intervention strategies with demonstrated effectiveness, and the creation of tools to support the widespread dissemination to and rapid implementation of intervention strategies in new settings. In real-world settings, such as organizations and clinics funded by the RWHAP, effective intervention strategies focus on reducing barriers and improving linkage to and retention in HIV care and adherence to medications, all of which lead to reaching and maintaining viral suppression for people with HIV.

HRSA HAB is working to achieve a balance between rigor and relevance in the development of intervention strategy toolkits and guidance manuals. Additionally, as HRSA HAB develops its centralized web portal to compile intervention strategies [42], we will conduct extensive stakeholder engagement to ensure that the intervention strategies disseminated are meeting the needs of RWHAP providers. Finally, in collaboration with our partners in the Federal Workgroup, HRSA HAB will continue to coordinate its own implementation science activities with those of other federal agencies, ensuring that we are complementing, rather than duplicating, each other’s efforts.

By maximizing the impact of the RWHAP for all clients, decreasing morbidity and mortality, and improving health outcomes along the HIV care continuum, implementation science is bringing us closer to ending the HIV epidemic in the US.

## Supporting information

S1 Appendix Applying the HAB IS framework in an existing cooperative agreement. HAB IS, HIV/AIDS Bureau implementation science approach.
(DOCX)

## Abbreviations:

CDC: Centers for Disease Control and Prevention
E2i: Using Evidence-Informed Interventions to Improve Health Outcomes among People Living with HIV
Federal Workgroup: Federal Implementation Science Workgroup
HAB: HIV/AIDS Bureau
HAB IS: HAB implementation science approach
HAB Workgroup: HAB Implementation Science Workgroup
HRSA: Health Resources and Services Administration
NIMH: National Institute of Mental Health
PEP: post-exposure prophylaxis
PrEP: pre-exposure prophylaxis
RCT: randomized controlled trial
RWHAP: Ryan White HIV/AIDS Program
SPNS: Special Projects of National Significance

## References

[1] Hargreaves J, Dalal S, Rice B, Anderegg N, Bhattacharjee P, Gafos M, Hensen B, Mangenah C, Quaife M, Padian N. Repositioning Implementation Science in the HIV Response: Looking Ahead From AIDS 2018. JAIDS Journal of Acquired Immune Deficiency Syndromes. 2019;1(82):S299–304. 10.1097/QAI.0000000000002209 31764267

[2] Eisinger RW, Dieffenbach CW, Fauci AS. Role of Implementation Science: Linking Fundamental Discovery Science and Innovation Science to Ending the HIV Epidemic at the Community Level. JAIDS Journal of Acquired Immune Deficiency Syndromes. 2019;1(82):S171–2. 10.1097/QAI.0000000000002227 31764250

[3] Underhill K, Operario D, Mimiaga MJ, Skeer MR, Mayer KH. Implementation science of pre-exposure prophylaxis: preparing for public use. Current HIV/AIDS Reports. 2010 11 1;7(4):210–9. 10.1007/s11904-010-0062-4 20820971 PMC3012127

[4] Glasgow RE, Eckstein ET, ElZarrad MK. Implementation science perspectives and opportunities for HIV/AIDS research: integrating science, practice, and policy. JAIDS Journal of Acquired Immune Deficiency Syndromes. 2013 6 1;63:S26–31. 10.1097/QAI.0b013e3182920286 23673882

[5] Schackman BR. Implementation science for the prevention and treatment of HIV/AIDS. Journal of acquired immune deficiency syndromes. 2010(1999);1(55)(1):S27 10.1097/QAI.0b013e3181f9c1da 21045596 PMC3058234

[6] Eccles MP, Mittman BS. Welcome to implementation science. 2006;1(1).

[7] Geng EH, Peiris D, Kruk ME. Implementation science: Relevance in the real world without sacrificing rigor. PLoS Med. 2017;14(4): e1002288 10.1371/journal.pmed.1002288 28441435 PMC5404833

[8] Rotheram-Borus MJ, Swendeman D, Chovnick G. The past, present, and future of HIV prevention: integrating behavioral, biomedical, and structural intervention strategies for the next generation of HIV prevention. Annual review of clinical psychology. 2009 4 27;5:143–67. 10.1146/annurev.clinpsy.032408.153530 19327028 PMC2864227

[9] Health Resources and Services Administration. Ryan White HIV/AIDS Program Annual Client-Level Data Report 2018. Available from: http://hab.hrsa.gov/data/data-reports. Published December 2019. [cited 2019 December 1].

[10] National HIV/AIDS Strategy for the United States: Updated to 2020. Available from: https://files.hiv.gov/s3fs-public/nhas-update.pdf. Published July 2015. [cited 2019 Mar 1].

[11] Mandsager P, Marier A, Cohen S, Fanning M, Hauck H, Cheever LW. Reducing HIV-related health disparities in the health resources and services administration’s Ryan White HIV/AIDS Program. American Journal of Public Health. 2018 11;108(S4):S246–50. 10.2105/AJPH.2018.304689 30383416 PMC6215373

[12] Ryan White HIV/AIDS Program and Incorporating Messages on the Impact of Viral Suppression. Available from: https://hab.hrsa.gov/sites/default/files/hab/program-grants-management/ViralSuppressionProgramLetterFinal10-19-2018.pdf. Published October 2018. [cited 2019 March 1].

[13] Molina JM, Ghosn J, Béniguel L, Rojas-Castro D, Algarte-Genin M, Pialoux G, Delaugerre C, Yazdanpanah Y, Katlama C, Ségouin C, Morel S. Incidence of HIV-infection in the ANRS Prévenir study in Paris region with daily or on-demand PrEP with TDF/FTC. 10.1080/09540121.2020.1742863 32174136

[14] Grant RM, Lama JR, Anderson PL, McMahan V, Liu AY, Vargas L, Goicochea P, Casapía M, Guanira-Carranza JV, Ramirez-Cardich ME, Montoya-Herrera O. Preexposure chemoprophylaxis for HIV prevention in men who have sex with men. New England Journal of Medicine. 2010 12 30;363(27):2587–99. 10.1056/NEJMoa1011205 21091279 PMC3079639

[15] Thigpen MC, Kebaabetswe PM, Paxton LA, Smith DK, Rose CE, Segolodi TM, Henderson FL, Pathak SR, Soud FA, Chillag KL, Mutanhaurwa R. Antiretroviral preexposure prophylaxis for heterosexual HIV transmission in Botswana. New England Journal of Medicine. 2012 8 2;367(5):423–34. 10.1056/NEJMoa1110711 22784038

[16] Baeten JM, Donnell D, Ndase P, Mugo NR, Campbell JD, Wangisi J, Tappero JW, Bukusi EA, Cohen CR, Katabira E, Ronald A. Antiretroviral prophylaxis for HIV prevention in heterosexual men and women. New England Journal of Medicine. 2012 8 2;367(5):399–410. 10.1056/NEJMoa1108524 22784037 PMC3770474

[17] Centers for Disease Control and Prevention. “Pre-Exposure Prophylaxis.” Available from: https://www.cdc.gov/hiv/risk/prep/index.html. [cited 2019 October 8].

[18] Fauci AS, Redfield RR, Sigounas G, Weahkee MD, Giroir BP. Ending the HIV Epidemic: A Plan for the United States. JAMA. 2019;321(9):844–845. 10.1001/jama.2019.1343 30730529

[19] Aarons GA, Hurlburt M, Horwitz SM. Advancing a conceptual model of evidence-based practice implementation in public service sectors. Administration and Policy in Mental Health and Mental Health Services Research. 2011 1 1;38(1):4–23. 10.1007/s10488-010-0327-7 21197565 PMC3025110

[20] Damschroder LJ, Aron DC, Keith RE, Kirsh SR, Alexander JA, Lowery JC. Fostering implementation of health services research findings into practice: a consolidated framework for advancing implementation science. Implement Sci. 2009;4:50 10.1186/1748-5908-4-50 19664226 PMC2736161

[21] Hirschhorn LR, Ojikutu B, Rodriguez W. Research for change: using implementation research to strengthen HIV care and treatment scale-up in resource-limited settings. The Journal of Infectious Diseases. 2007;1;196(3):S516–22. 10.1086/521120 18181704

[22] Glasgow RE, Vogt TM, Boles SM. Evaluating the public health impact of health promotion interventions: the RE-AIM framework. American Journal of Public Health. 1999 9;89(9):1322–7. 10.2105/ajph.89.9.1322 10474547 PMC1508772

[23] Proctor E. K., Landsverk J., Aarons G., Chambers D., Glisson C., & Mittman B. (2009). Implementation research in mental health services: an emerging science with conceptual, methodological, and training challenges. Administration and Policy in Mental Health and Mental Health Services Research, 36(1), 24–34. 10.1007/s10488-008-0197-4 19104929 PMC3808121

[24] Green L, Kreuter M. The precede–proceed model Health promotion planning: an educational approach. 3rd ed. Mountain View (CA): Mayfield Publishing Company 1999:32–43.

[25] Feldstein AC, Glasgow RE. A practical, robust implementation and sustainability model (PRISM) for integrating research findings into practice. The Joint Commission Journal on Quality and Patient Safety. 2008 4 1;34(4):228–43. 10.1016/s1553-7250(08)34030-6 18468362

[26] Rabin BA, Brownson RC. Terminology for dissemination and implementation research. Dissemination and implementation research in health: Translating science to practice. 2017 11 10;2:19–45.

[27] Brown CH, Curran G, Palinkas LA, Aarons GA, Wells KB, Jones L, Collins LM, Duan N, Mittman BS, Wallace A, Tabak RG. An overview of research and evaluation designs for dissemination and implementation. Annual Review of Public Health. 2017; 20(38):1–22. 10.1146/annurev-publhealth-031816-044215 28384085 PMC5384265

[28] Proctor EK, Powell BJ, McMillen JC. Implementation strategies: recommendations for specifying and reporting. Implementation Science. 2013 12;8(1):139 10.1186/1748-5908-8-139 24289295 PMC3882890

[29] 6.Michie S, Fixsen DL, Grimshaw JM, Eccles MP. Specifying and reporting complex behaviour change interventions: the need for a scientific method. Implement Science. 2009;4:1–6. 10.1186/1748-5908-4-40 19607700 PMC2717906

[30] 9.Waltz TJ, Powell BJ, Chinman MJ, Smith JL, Matthieu MM, Proctor EK, et al Expert Recommendations for Implementing Change (ERIC): protocol for a mixed methods study. Implement Science. 2014;9:1–12. 10.1186/1748-5908-9-39 24669765 PMC3987065

[31] Mitchell SA, Fisher CA, Hastings CE, Silverman LB, Wallen GR. A thematic analysis of theoretical models for translating science in nursing: mapping the field. Nursing Outlook. 2010;58:287–300. 10.1016/j.outlook.2010.07.001 21074646 PMC3011939

[32] ICEBeRG. Designing theoretically-informed implementation interventions. Implement Sci. 2006;1:4 10.1186/1748-5908-1-4 16722571 PMC1436012

[33] Martinez RG, Lewis CC, Weiner BJ. Instrumentation issues in implementation science. Implement Sci. 2014;9:118 10.1186/s13012-014-0118-8 25185799 PMC4164742

[34] Nilsen P. Making sense of implementation theories, models and frameworks. Implementation Science. 2015 12;10(1):53 10.1186/s13012-015-0242-0 25895742 PMC4406164

[35] Sturke R, Harmston C, Simonds RJ, Mofenson LM, Siberry GK, Watts DH, et al A multi-disciplinary approach to implementation science: the NIH-PEPFAR PMTCT implementation science alliance. Journal of Acquired Immune Deficiency Syndromes. 2014; 67(2):S163±7. 10.1097/QAI.0000000000000323 25310124

[36] H Lambdin B, Cheng B, Peter T, Mbwambo J, Apollo T, Dunbar M, C Udoh I, Cattamanchi A, H Geng E, Volberding P. Implementing implementation science: an approach for HIV prevention, care and treatment programs. Current HIV Research. 2015;13(3):244–6. 10.2174/1570162x1303150506185423 25986374 PMC4460284

[37] Mark D, Geng E, Vorkoper S, Essajee S, Bloch K, Willis N, Stewart B, Bakeera-Kitaka S, Sugandhi N, Sturke R, Achebe K. Making implementation science work for children and adolescents living with HIV. Journal of Acquired Immune Deficiency Syndromes (1999). 2018;78(1):S58 10.1097/QAI.0000000000001750 29994921 PMC6044463

[38] Sturke R, Siberry G, Mofenson L, Watts DH, McIntyre JA, Brouwers P, Guay L. Creating sustainable collaborations for implementation science: the case of the NIH-PEPFAR PMTCT Implementation Science Alliance. JAIDS Journal of Acquired Immune Deficiency Syndromes. 2016 8 1;72:S102–7. 10.1097/QAI.0000000000001065 27355496

[39] AIDS United. Ending the HIV Epidemic in the United States: A Roadmap for Federal Action. Released November 30, 2018. Available from: http://www.aidsunited.org/data/files/Site_18/Policy/Ending_the_HIV_Epidemic_U.S._Roadmap_for_Federal_%20Action_FINAL.pdf. [cited 2019 Oct 12].

[40] 10. Curran G. M., Bauer M., Mittman B., Pyne J. M., & Stetler C. (2012). Effectiveness-implementation hybrid designs: combining elements of clinical effectiveness and implementation research to enhance public health impact. Medical Care, 50(3), 217 10.1097/MLR.0b013e3182408812 22310560 PMC3731143

[41] E2i: Using Evidence-Informed Interventions to Improve Health Outcomes among People Living with HIV. Available from: https://targethiv.org/e2i. [cited 2020 Jan 3].

[42] RWHAP Best Practices Recipient Compilation. TargetHIV. Available from: https://targethiv.org/ta-org/rwhap-best-practices-recipient-compilation. [cited 2020 Jan 3].

