# Supplementary material for: Implementation science and the Health Resources and Services Administration’s Ryan White HIV/AIDS Program’s work towards ending the HIV epidemic in the United States
Source: PLoS Med. 2020 Nov 6;17(11):e1003128. doi: 10.1371/journal.pmed.1003128 (PMC7647058; doi:10.1371/journal.pmed.1003128)
Supplement: S1 Appendix — HAB IS, HIV/AIDS Bureau implementation science approach. (DOCX) [file pmed.1003128.s001.docx]

| **RAPID IMPLEMENTATION** | **USING EVIDENCE-INFORMED INTERVENTIONS TO IMPROVE OUTCOMES FOR PEOPLE LIVING WITH HIV (E2i) (2017-2021)** |  |
| --- | --- | --- |
| **Identify gaps in care using program data** | Ryan White HIV/AIDS Program (RWHAP) client-level data from 2010-2016 informed the identification of four focus areas of high need: 1) improving HIV health outcomes for transgender women, 2) improving HIV health outcomes for Black men who have sex with men, 3) integrating behavioral health with primary medical care for people with HIV, and 4) identifying and addressing trauma among people with HIV. |  |
| **Identify existing intervention strategies** | The E2i Center for Technical Assistance (CCTA), in consultation with Health Resources and Services Administration’s HIV/AIDS Bureau (HRSA HAB), identified existing interventions by searching online medical databases as well as the websites of academic research centers, non-profit groups, and federal agencies. Additional recommendations and unpublished materials were then solicited from subject matter experts and intervention developers. |  |
| **Determine appropriate assessment criteria** | The CCTA, in consultation with HRSA HAB, developed a quantitative scoring rubric to assess the identified interventions for quality of evidence, relevance to the focus area, cultural appropriateness for priority populations, and feasibility for RWHAP-funded sites. In addition, the rubric includes qualitative open-ended questions on the strengths and weaknesses of each intervention. |  |
| **Assess demonstrated effectiveness** | Across the four focus areas, 44 interventions were determined to have sufficient quality of documentation to be routed to the evidence-informed interventions rubric. Subject matter experts and community members scored the interventions and then met in-person to discuss the top-ranked interventions for each focus area. Ultimately, four interventions per focus area were selected as most promising for implementation. |  |
| **Identify core elements** | The CCTA, in consultation with the developers of the interventions and HRSA HAB, created a logic model to identify the core elements for each of the interventions. |  |
| **Tailor/adapt for RWHAP** | The CCTA, in consultation with HRSA HAB, provided technical assistance to the 26 sites with extensive support from the intervention developers to tailor the interventions for implementation in their cultural and organizational contexts. Although the core elements of the interventions could not be altered, the sites were encouraged and supported to identify implementation strategies to further tailor the interventions for successful implementation in their setting. |  |
| **Implement and evaluate at pilot sites** | A competitive request for proposals solicited applications from RWHAP-funded sites to implement one of the top four interventions in one E2i focus area. Applications were ranked according to need, capacity, implementation plan, and other factors. Twenty-six sites were selected, and 11 interventions are being implemented. Technical assistance to support implementation is being provided by the CCTA. |  |
| **Develop intervention implementation toolkits** | Multimedia implementation toolkits are being developed and piloted with the RWHAP sites. Designed to be accessible for a broad audience, the toolkits will help facilitate rapid implementation of the interventions in the future without the need for extensive technical assistance. |  |
| **Disseminate toolkits for rapid replication** | Through TargetHIV.gov, HRSA HAB will disseminate interventions found to have been successfully implemented, with associated positive impacts on health outcomes for clients engaged in the intervention. |  |
| **Assess uptake and impact** | Through ongoing technical assistance activities and program monitoring, HRSA HAB will assess the uptake/successful replication of interventions centrally disseminated though TargetHIV.gov, and gauge their associated impact on RWHAP client outcomes. |  |
| *Note: The he Fenway Institute, in partnership with AIDS United, is serving as the Center for Technical Assistance* | | |
| *(CCTA).* | | |
| **IMPLEMENTATION SCIENCE EVALUATION** | **USING EVIDENCE-INFORMED INTERVENTIONS TO IMPROVE OUTCOMES FOR PEOPLE LIVING WITH HIV (E2i) (2017-2021)** |  |
| **Evaluate implementation at select RWHAP sites** | The E2i Evaluation Center (EC), in consultation with HRSA HAB, adapted the Proctor Model to develop a comprehensive, multi-site, mixed-methods evaluation. The evaluation uses qualitative methods to document intervention strategies, quantitative methods to assess implementation and client health outcomes, and mixed methods for a comprehensive understanding of implementation strategies used and outcomes. The evaluation was designed to minimize data collection burden on intervention staff and requires no collection directly from participants |  |
| **Barriers/ facilitators** | During a 3-month formative phase prior to intervention implementation, initial program documents at the 26 sites were reviewed. Additionally, sites completed a baseline organizational assessment to further gauge readiness to implement. |  |
| **Intervention strategies** | Secondary document review is being used to assess intervention uptake and mid-course changes to intervention delivery. Intervention exposure is measured through intervention enrollment forms completed upon each encounter with E2i participants; this contributes to the evaluation of the degree to which the intervention was implemented (uptake) and implemented as intended (fidelity). |  |
| **Implementation strategies** | The 26 sites proposed a specific implementation plan that included implementation strategies. However, sites were also encouraged to adjust those implementation strategies if necessary. Biannual learning sessions including all 26 sites and monthly monitoring calls are being used to assess implementation progress, challenges, and processes, with particular attention paid to fidelity to site's original implementation plan and any mid-implementation adjustments to implementation strategies. |  |
| **Implementation outcomes** | The evaluation is addressing the 3 key measures identified in the HRSA HAB IS evaluation framework through a mixed-methods adaptation of the Proctor Model. In addition to uptake, engagement (penetration), and barriers/facilitators (feasibility, acceptability, sustainability), the evaluation is measuring fidelity, cost, and 4 service outcomes (efficiency, effectiveness, equity, timeliness). Observations conducted during biannual learning sessions and secondary document review will be structured to capture site perspectives on implementation outcomes. Organizational assessments are being conducted every 6 months to assess organizational contextual changes and outcomes (barriers/facilitators). Finally, annual cost assessments will measure the financial and human capital resources directed toward the implementation of the interventions. This will permit an assessment of the cost per person of implementing the intervention (cost, efficiency). |  |
| **Client HIV outcomes** | Information about HIV health outcomes, particularly viral suppression, for clients engaged in the interventions, are collected every 6 months, from 12 months prior to and 12 months following enrollment in the intervention. Sites use a combination of Ryan White Service Report data or abstracted data collected from medical records to report de-identified outcomes to the evaluation team through a secure portal. |  |
| *Note: The University of California, San Francisco's Center for AIDS Prevention Studies is serving as the* | | |
| *Evaluation Center (EC).* | | |
